# High-resolution mapping of spatial heterogeneity in ice wedge polygon geomorphology near Prudhoe Bay, Alaska

**DOI:** 10.1038/s41597-020-0423-9

**Published:** 2020-03-10

**Authors:** Charles J. Abolt, Michael H. Young

**Affiliations:** 1 Department of Geological Sciences, The University of Texas at Austin, Austin, TX USA; 2 Bureau of Economic Geology, The University of Texas at Austin, Austin, TX USA; 3 Present Address: Earth and Environmental Sciences Division, Los Alamos National Laboratory, Los Alamos, NM USA

**Keywords:** Hydrology, Environmental sciences, Cryospheric science

## Abstract

It is well known that microtopography associated with ice wedge polygons drives pronounced, meter-scale spatial gradients in hydrologic and ecological processes on the tundra. However, high-resolution maps of polygonal geomorphology are rarely available, due to the complexity and subtlety of ice wedge polygon relief at landscape scales. Here we present a sub-meter resolution map of >10^6^ discrete ice wedge polygons across a ~1200 km^2^ landscape, delineated within a lidar-derived digital elevation model. The delineation procedure relies on a convolutional neural network paired with a set of common image processing operations and permits explicit measurement of relative elevation at the center of each ice wedge polygon. The resulting map visualizes meter- to kilometer-scale spatial gradients in polygonal geomorphology across an extensive landscape with unprecedented detail. This high-resolution inventory of polygonal geomorphology provides rich spatial context for extrapolating observations of environmental processes across the landscape. The map also represents an extensive baseline dataset for quantifying contemporary land surface deformation (*i.e*., thermokarst) at the survey area, through future topographic surveys.

## Abstract (summary)

**Table:** 

Measurement(s)	ice wedge polygon • land
Technology Type(s)	computational modeling technique • Lidar
Sample Characteristic - Environment	tundra
Sample Characteristic - Location	State of Alaska

Machine-accessible metadata file describing the reported data: 10.6084/m9.figshare.11871369

## Background & Summary

One of the challenges to upscaling hydrologic and ecological measurements in tundra settings is accounting for spatial heterogeneity associated with ice wedge polygons. Segmenting the ground into discrete units some tens of meters across, ice wedge polygons are some of the most conspicuous features of the tundra surface. However, mapping ice wedge polygons across broad spatial scales is challenging, due to the subtlety and complexity of polygonal microtopography. Commonly, features demarcating the edges of polygons—such as rims of soil in low-centered polygons (LCPs), or sunken troughs in high-centered polygons (HCPs)—represent less than 50 cm of relief from the surrounding terrain^1,2^. Nonetheless, variability in this microtopography often drives sharp spatial gradients in fluxes of energy^3–5^, water^6,7^, and carbon^8–11^ between the atmosphere and the subsurface. The spatial distribution of polygons of different morphologies within a single landscape can be highly complex, as thousands of polygons may occupy a single square kilometer of terrain, and gradation between endmembers (such as LCPs and HCPs) may occur over distances that vary from meters to kilometers^12,13^. The difficulty of accounting for the influence of such complexity on environmental processes is compounded by the transient nature of the microtopography itself, which can deform over timescales of years to decades in response to increased ground temperatures^6,14–19^. High-resolution inventories of spatial and temporal heterogeneity in ice wedge polygon form are therefore important for quantifying and predicting future trends in landscape-scale processes, including mobilization of soil organic carbon, which may exert important feedbacks on global climate change over the coming century^20^.

To date, most efforts to map ice wedge polygon morphology have relied on optical or multispectral imagery, and have been designed to classify terrain into distinct bins, such as LCPs, HCPs, or intermediate polygons (*e.g*.^6,10,12,14,15,17,21^). The spatial extent of previous maps has varied from ~1 km^2^ or less, to the entirety of the Alaska North Slope. These landcover maps play an essential role in dividing the Arctic into functional terrain units, for extrapolating point- to plot-scale measurements of hydrologic and ecological processes across landscapes. However, to our knowledge, no dataset yet published quantifies gradational (*i.e*., non-binned) change in polygonal morphology over spatial scales greater than several square kilometers. This gap prevents quantitative assessment of the degree of heterogeneity within each polygonal class, and also limits the precision with which contemporary rates of land surface evolution in polygonal terrain can be quantified.

Here we present a high-resolution map of polygonal morphology across a ~1,200 km^2^ landscape south of Prudhoe Bay, Alaska, USA. Analyzing a sub-meter resolution digital elevation model derived from a set of airborne lidar surveys, we delineate >10^6^ individual polygons and explicitly measure the microtopography of each, permitting assignment along a continuous spectrum between LCP and HCP forms. Providing a snapshot in time from the period of data acquisition, the dataset offers an unprecedented quantitative summary of the extent to which polygonal morphology varies within a single landscape across distances of meters to tens of kilometers. Additionally, the map comprises an extensive baseline dataset for quantifying contemporary rates of land surface evolution, through comparison with future topographic surveys.

## Methods

### Study area and data acquisition

**Fig. 1:**
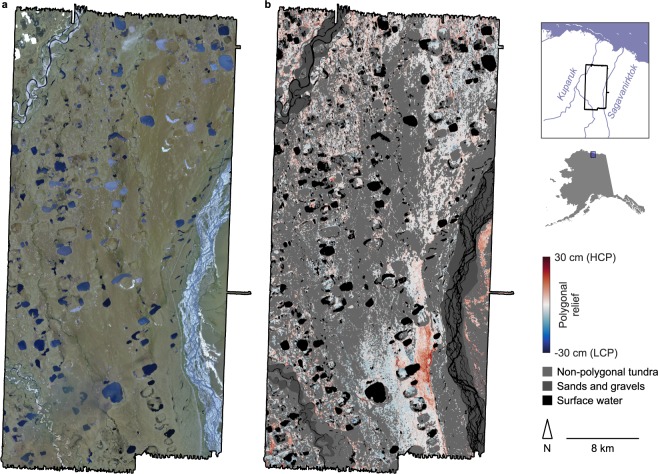
Visual imagery and results from the full lidar survey area. (**a**) Orthorectified imagery of the landscape from the SPOT-5 satellite^31^. (**b**) Map of polygonal geomorphology, with extents of coarse alluvial sands and gravels and surface water imposed.

The airborne lidar survey area comprises 1210 km^2^ in northern Alaska, USA, centered ~60 km south of Prudhoe Bay (Fig. 1a). The site is roughly bounded by the Sagavanirktok River to the east and the Kuparuk River to the west, and falls within the Arctic peaty lowlands ecological landscape, which occupies approximately half of the Arctic Coastal Plain in Alaska^22^. Soil pits excavated in the region indicate that surface sediments throughout most of the site are capped with ~1–3 m of Pleistocene-aged aeolian silts, grading upward into a mantle of peat some tens of centimeters thick, which has developed over the last several thousand years^23,24^. These silts overlie several meters of coarse alluvial sands and gravels, deposited by ancient braided streams carrying sediments northward from the Brooks Range mountains^17^. Typifying the broader coastal plain, the study area contains hundreds of shallow thaw lakes, generally less than 5 m deep, and ranging in area from ~1 ha to 1 km^2^. Interspersed among these modern lakes, the site also contains >100 instances of ancient thaw lake basins that have drained in the last 10,000 years, leaving shallow valleys in the modern land surface, in which the original fluvial sediments often are reworked and capped with lacustrine silts^25^. Throughout the survey area, vegetation is generally low-lying and homogeneous, consisting primarily of grasses and sedges.

The input to our procedure for mapping ice wedge polygons in the survey area was a high-resolution digital elevation model (DEM), derived from a set of two airborne lidar surveys, one conducted during August 2012 and the other during August 2014. The primary laser pulsing system on the lidar instrument (Chiroptera, Airborne Hydrography AB) operated within the near-infrared spectrum (1064 nm) at a frequency of 400 kHz (a second pulsing system fired at 38 kHz operated at 515 nm to measure bathymetric profiles of surface water bodies). Deployed in an aircraft at a nominal altitude of 400 m, it generated a point cloud representation of the land surface with an average return density greater than 20 points m^−2^ ^26^. Vertical accuracy of the point cloud was determined by comparing airborne lidar returns with a ground-based kinematic GPS survey of the runway at the Deadhorse, Alaska airport; at 156 ground control points, the average bias was −0.001 m, average absolute error was 0.023 m, and the maximum absolute error was 0.075 m. Horizontal and vertical precision in the remainder of the surveyed area was determined by analyzing offsets between adjacent and overlapping flight lines; >95% of errors in both orientations were 3 cm or less. For use in our workflow, point cloud data were rasterized at 50 cm horizontal resolution.

In addition to the DEM, our procedure employed two secondary geospatial datasets, demarcating zones of the survey area in which either open water or exposed sands and gravels associated with the Sagavanirktok and Kuparuk Rivers were present at the surface. These data were used to indicate zones of the DEM in which our algorithm was instructed not to delineate polygonal ground, as ice wedges would not be expected to form in such terrain. Landcover by both water and coarse sands and gravels were mapped as 50 cm horizontal resolution rasters, snapped to the original DEM. The landcover raster representing water was generated prior to this study by extracting zones of divergence between elevation as estimated by the red and green lasers on the lidar instrument^26^. Landcover by gravels and sands was mapped by hand atop Google Earth imagery by drawing polygons surrounding exposed bars adjacent to the two major streams of the survey area, which were distinctly brighter than the dark green to brown tones of the nearby tundra soils. These polygons were subsequently converted into rasters, snapped to the original DEM, for use during mapping.

### Delineation of ice wedge polygons

Ice wedge polygons were delineated within the DEM using a novel, deep learning-based approach^13^, which is briefly summarized here. At its core, the method relied on a convolutional neural network (CNN) to infer whether or not each pixel of the DEM represents a polygon boundary. The binary map of polygon boundaries produced by the CNN was then processed to segment the DEM into discrete polygons. (All data and code necessary to repeat our procedure are presented with the final dataset).

In the first stage of the workflow, mesoscale topographic trends were removed from the DEM, which was then converted into 8-bit grayscale imagery, in preparation for processing by the CNN. Mesoscale topography was first estimated by applying a two-dimensional averaging filter across the DEM with a radius of 20 m (*i.e*., a distance comparable to the width of a typical ice wedge polygon). This mesoscale topography was subtracted from the DEM to isolate microtopography associated with individual polygons. The raster of microtopographic data was then converted into a grayscale image, in which local peaks >0.7 m in relief were assigned a maximum brightness, and pits >0.7 m in depth were assigned minimum brightness. These bounds were chosen to enforce a consistent relationship between image intensity and microtopographic relief, while capturing the vast majority of microtopography encountered on the tundra.

Our CNN was designed to operate on the microtopographic imagery one pixel at a time, generating a binary label as “boundary” or “not boundary.” For each pixel, a thumbnail image of the surrounding 27 × 27 pixel neighborhood in the 8-bit microtopographic imagery was used as input (*i.e*., 100 overlapping thumbnail images, each 27 × 27 pixels in area, would be generated and passed to the CNN to process each 10 × 10 pixel subset of the DEM). The CNN was purposefully designed using a simple architecture (Table 1), which permitted efficient training and execution. Training was conducted on a stack of 71,930 thumbnail images, evenly divided into “boundary” and “not boundary” examples, derived from manually labeled imagery representing ~14.5 ha of terrain, or ~0.015% of the survey area. Training was conducting using a standard stochastic gradient descent approach to calibrate the weights and biases of the CNN. The training procedure was executed on a personal laptop with a single NVIDIA MX150 graphic processing unit (GPU), and required less than 30 minutes to achieve >98% accuracy. Subsequently, application of the trained CNN across the survey area was executed on an HPC cluster at the Texas Advanced Computing Center using four NVIDIA K-40 GPUs. Approximately eight hours of processing time were required to generate a binary map of the entire ~1200 km^2^ landscape.

**Table 1:** Architecture of CNN.

Layer	Type	Neurons
1	Convolutional	16 arrays of 27 × 27
2	ReLU^†^	16 arrays of 27 × 27
3	Max-pooling	16 arrays of 9 × 9
4	Convolutional	256 arrays of 9 × 9
5	ReLU	256 arrays of 9 × 9
6	Fully-connected	64
7	ReLU	64
8	Fully-connected	2
9	ReLU	2
10	Softmax	2

^†^ReLu – rectified linear unit.

Following application of the CNN, the binary map of polygon boundaries was processed in several stages to extract discrete polygons. In the first stage, all continuous regions identified as “boundary” but with an area less than 20 m^2^ were eliminated, in an effort to remove false positives. This threshold was chosen based on the rationale that any pixel representing a true ice wedge polygon boundary would be expected to comprise part of a continuous network, arbitrarily larger than 20 m^2^ in area. In the second stage, the Euclidean distance from the closest boundary of each “non-boundary” pixel was calculated and multiplied by −1 (*i.e*., a distance transform was applied), generating an intermediate raster in which the area of an individual polygon comprised a valley of negative signal intensity. At this stage, to minimize the likelihood of over-segmentation, any local valley with a minimum depth <1.5 m was fused with the nearest larger valley using morphological reconstruction^27^. This operation was based on the rationale that most real instances of ice wedge polygons contain at least one point greater than 1.5 m in distance from the closest boundary. Subsequently, a watershed transform was used to delineate the divides between valleys, thereby segmenting the intermediate raster into discrete regions, each representing a potential ice wedge polygon.

In the final stages of processing, each edge in the segmented image (*i.e*., each string of pixels on the divide between two adjacent regions) was analyzed individually. Any edge along which less than half of the pixels had been recognized as an ice wedge polygon boundary by the CNN was eliminated, and the adjacent regions were merged. Effectively, this procedure tended to preserve the boundaries of real ice wedge polygons, while lumping non-polygonal terrain into regions much larger than a typical polygon. Non-polygonal terrain was then removed from the final raster by eliminating all regions greater than 10,000 m^2^, a threshold which was chosen to be greater than the area of a real ice wedge polygon. Finally, any remaining polygons delineated by the algorithm overlapping surface water or the coarse sediments of the Sagavanirktok and Kuparuk rivers were treated as false positives and removed. Typically, all post-processing subsequent to the application of the CNN required 15 seconds or fewer per square kilometer.

### Measurement of microtopography

**Fig. 2:**
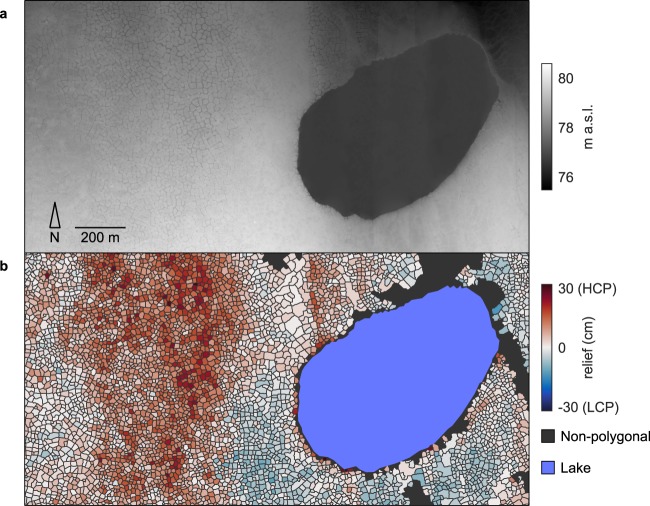
DEM and results from a 2 km^2^ subset of the survey area. (**a**) 50 cm horizontal resolution DEM. (**b**) Map of polygonal geomorphology.

Subsequent to delineation, we measured the elevation at each ice wedge polygon center relative to the periphery, as a proxy for low-centeredness or high-centeredness. The procedure we employed was identical to that presented in^13^. For each polygon, a distance transform was first applied to calculate the distance of each pixel within the polygonal area from the closest boundary. The median of these calculated distances was then used to split the polygonal area into two subregions: a ring of outer pixels, closest to the boundaries, and an equally sized core of inner pixels. The mean elevation of the outer subregion, calculated using data from the DEM, was then subtracted from the mean elevation in the polygon interior. Conceptually, this procedure provided a simple and deterministic means for placing each polygon along a spectrum that varied from low-centered to high-centered. Executed on an NVIDIA K-40 GPU, this procedure required ~30–45 seconds per square kilometer, depending on the number of polygons present. The results permit high-resolution visualization of gradation in polygon geomorphology, at spatial scales varying from tens of kilometers (Fig. 1b) to meters (Fig. 2).

### Vectorization of polygon boundaries

Our method of delineating ice wedge polygons, as described above, produced labeled raster images in which each ice wedge polygon was assigned a unique ID. For greater usability, we subsequently vectorized polygon boundaries, which were saved in shapefile format. Our vectorization procedure was designed to operate individually on each edge, or line of pixels, dividing two adjacent polygons. A polyline was first defined by connecting the center points of the pixels along an edge. Jagged segments along the polyline were then smoothed by applying an algorithm which removed non-endpoint vertices one at a time, simplifying the polyline as far as possible without permitting any point along its distance to diverge more than one meter from the original edge. Repeated along every edge in the labeled raster, this procedure defined the boundaries of an equivalent set of interlocking polygons.

## Data Records

The final map and ancillary datasets have been uploaded to a PANGAEA repository^28^. The map of polygon boundaries is presented in ESRI shapefile format, projected to UTM Zone 6N coordinates using the WGS84 geographic datum. Individual shapefiles correspond to 1 km^2^ subsets of the survey area, containing all ice wedge polygons with centroids located in that subset. The attribute table of a shapefile includes the unique ID of each polygon, the subset of the survey area in which it is located, area (m^2^), UTM coordinates of the polygon centroid (m), and relative elevation at the polygon center (m). A single tab-delineated file summarizing these attributes across the entire survey area is also included.

All rasters included with the dataset are presented in geotiff format at 50 cm horizontal resolution. For usability, rasters are also broken into multiple files corresponding to 1 km^2^ subsets of the survey area. The DEM used as input to the mapping procedure is presented as a single band raster with units of meters above mean sea level, in double-precision floating-point format. The raster of microtopographic imagery is presented as single-band, 8-bit grayscale imagery. Additionally, the full resolution lidar point cloud used to create the DEM is available in LAZ format.

Besides the DEM and microtopography, two additional rasters are included with the dataset: a labeled raster of the unique IDs for each polygon, and raw output from the convolutional neural network. These rasters are provided for use with the script for manual validation of results. The raster of unique IDs is presented as a set of single-band, 16-bit, labeled images, in which the pixels representing each identified polygon are assigned a unique integer label. Because some polygons straddle the divisions between adjacent square kilometer regions, each labeled raster contains a 100 m (200 pixel) buffer around the edges. The raster of raw CNN output is presented as a binary image in which pixels recognized as polygon boundaries are labeled “true.”

Finally, code and instructions for repeating the procedures for CNN training, polygon delineation, measurement of microtopography, technical validation, and vectorization of polygon boundaries are included in a repository accompanying the main dataset.

## Technical Validation

Accuracy of the polygonal delineation procedure was determined through manual validation of the results. Of the 1.16 × 10^6^ polygons identified in the survey area, 1200 (~0.1%) were selected randomly for this procedure. One at a time, the boundaries of a polygon were projected atop the 8-bit microtopographic imagery and visually inspected. Each polygon was classified as whole, fragmentary, conglomerate, or false. For this analysis, a fragmentary polygon was defined as a machine-delineated polygon including less than 90% of the true area of an ice wedge polygon, as determined by the observer. Conglomerates were defined as polygons comprising portions of multiple ice wedge polygons. This definition includes some ambiguity, as field observations indicate that ice wedge polygons are often subdivided into secondary and tertiary polygons, associated with relatively recent ice wedge cracking^29,30^. In general, to maintain consistency, an ice wedge polygon that appeared to contain subdivisions was labeled as conglomerate if any of the interior boundaries, discernible to the inspector, were more prominent than the exterior boundaries of the polygon delineated by the algorithm. Finally, a polygon delineated by the algorithm was classified as false if it occupied terrain in which no ice wedge polygon network was discernible to the observer.

Overall, the manual validation revealed that the delineation algorithm was highly skilled. Using the above criteria, 91% of polygons were classified as whole (Table 2). Among errors, fragments (~5%) and conglomerates (~4%) occurred with nearly equal frequency, while instances of false polygons (<1%) were rare.

**Table 2:** Results from manual validation procedure.

	Whole	Fragment	Conglomerate	False	Total
Instances	1092 (91%)	56 (5%)	44 (4%)	8 (<1%)	1200
Area (m^2^)	68897 (84%)	2452 (3%)	10295 (12%)	749 (1%)	82393

While completing the validation procedure, we observed rare instances of well-defined polygons (discernible to the inspector) which the algorithm failed to identify. These cases tended to be limited to terrain with unusually subtle microtopographic relief. Specifically, most instances were located within the drained basins of former lakes, some of which were characterized by large, flat polygons demarcated by narrow and shallow troughs. We attribute this mistake to the faintness of the microtopography associated with such polygons, rendering the boundaries difficult for the algorithm to distinguish from other local depressions on the tundra surface. We also note that, among terrain mapped as non-polygonal tundra within our datasets, scattered evidence of ice wedge-related trough development was commonly observed. However, trough segments in the affected regions failed to form a coherent network dividing the surface into discrete polygons. These isolated troughs indicate that: (1) the fraction of ground surface explicitly covered by polygonal microtopography is only a subset of the terrain affected by ice wedges; and (2) HCP formation among previously non-polygonal terrain may be incipient in many zones across the survey area, reflecting a trajectory of land surface evolution that recently has been observed in other landscapes underlain by massive ground ice, where no polygonal microtopography was discernible at the surface only years ago (*e.g*.^6^). In this context, we anticipate that future topographic measurements at the survey area may reveal greater coverage by discrete ice wedge polygons than is visible in our datasets from 2012–2014.

## Data Availability

All data and code necessary to replicate each stage of the mapping procedure, including training the CNN, mapping polygons, measuring microtopography, and validating results, is included in the same repository as the final data sets^28^. All code was written in MATLAB (version R2017b), and uses the Image Processing, Mapping, Neural Network, and Parallel Computing toolboxes.
